# Using genetic relatedness to understand heterogeneous distributions of urban rat‐associated pathogens

**DOI:** 10.1111/eva.13049

**Published:** 2020-07-23

**Authors:** Kaylee A. Byers, Tom R. Booker, Matthew Combs, Chelsea G. Himsworth, Jason Munshi‐South, David M. Patrick, Michael C. Whitlock

**Affiliations:** ^1^ Department of Interdisciplinary Studies University of British Columbia Vancouver BC Canada; ^2^ Biodiversity Research Centre University of British Columbia Vancouver BC Canada; ^3^ Canadian Wildlife Health Cooperative Animal Health Centre British Columbia Ministry of Agriculture Abbotsford BC Canada; ^4^ Department of Ecology, Evolution and Environmental Biology Columbia University New York NY USA; ^5^ School of Population and Public Health University of British Columbia Vancouver BC Canada; ^6^ Animal Health Centre British Columbia Ministry of Agriculture Abbotsford BC Canada; ^7^ Louis Calder Center‐Biological Field Station and Department of Biological Science Fordham University Armonk NY USA; ^8^ British Columbia Centre for Disease Control Vancouver BC Canada

**Keywords:** *Bartonella tribocorum*, *Clostridium difficile*, *Leptospira interrogans*, movement, Norway rat, parentage, pathogen, urban

## Abstract

Urban Norway rats (*Rattus norvegicus*) carry several pathogens transmissible to people. However, pathogen prevalence can vary across fine spatial scales (i.e., by city block). Using a population genomics approach, we sought to describe rat movement patterns across an urban landscape and to evaluate whether these patterns align with pathogen distributions. We genotyped 605 rats from a single neighborhood in Vancouver, Canada, and used 1,495 genome‐wide single nucleotide polymorphisms to identify parent–offspring and sibling relationships using pedigree analysis. We resolved 1,246 pairs of relatives, of which only 1% of pairs were captured in different city blocks. Relatives were primarily caught within 33 meters of each other leading to a highly leptokurtic distribution of dispersal distances. Using binomial generalized linear mixed models, we evaluated whether family relationships influenced rat pathogen status with the bacterial pathogens *Leptospira interrogans*, *Bartonella tribocorum*, and *Clostridium difficile*, and found that an individual's pathogen status was not predicted any better by including disease status of related rats. The spatial clustering of related rats and their pathogens lends support to the hypothesis that spatially restricted movement promotes the heterogeneous patterns of pathogen prevalence evidenced in this population. Our findings also highlight the utility of evolutionary tools to understand movement and rat‐associated health risks in urban landscapes.

## 1 INTRODUCTION

Norway rats (*Rattus norvegicus*) are carriers of a number of “zoonotic” pathogens (those transmissible between animals and humans) responsible for significant morbidity and mortality in cities globally (Himsworth, Parsons, et al., 2014). For example, the rat‐associated pathogen *Leptospira interrogans* affects approximately one million people annually and can result in kidney failure or pulmonary hemorrhage (Costa, Hagan, et al., 2015; Guerra, 2009). Urban rats serve as reservoirs for numerous important pathogens including *Yersinia pestis*, *Bartonella* spp., *Rickettsia typhi,* and Seoul hantavirus (Easterbrook et al., 2007; Firth et al., 2014; Himsworth, Parsons, Jardine, & Patrick, 2013; Pépin, 2016). With the exception of *Y. pestis* (the etiologic agent of plague), these pathogens are not known to cause any associated disease in rats (Himsworth, Parsons, et al., 2013). In addition, rats can carry human‐associated pathogens such as methicillin‐resistant *Staphylococcus aureus* (MRSA) (Himsworth, Miller, et al., 2014) and *Clostridium difficile* (Himsworth, Patrick, et al., 2014), although whether their carriage contributes to human transmission is unknown. Rat‐associated pathogens are spread among rats and to people in various ways, including through direct contact with rats, via disease vectors (i.e., fleas and lice), and through environmental contamination with rat urine and/or feces (Himsworth, Parsons, et al., 2013). Understanding rat‐pathogen dynamics is an increasingly important issue internationally given the global distribution of rats (Long, 2003) and rapid urbanization and densification of cities (United Nations, 2018) which is likely to intensify rat‐associated impacts worldwide (Himsworth, Parsons, et al., 2013).

**FIGURE 1:**
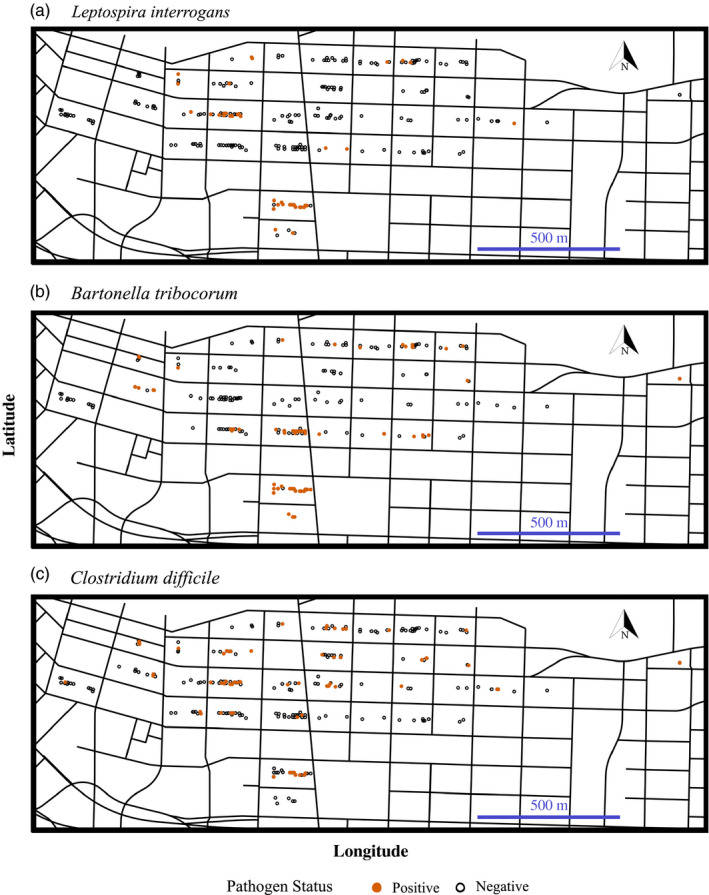
Spatial distribution of Norway rats (*Rattus norvegicus*) carrying pathogens across Vancouver's Downtown Eastside neighborhood. Rats were tested for carriage with: (a) *Leptospira interrogans* of which 11% (60/535) tested positive; (b) *Bartonella tribocorum* of which 26% (90/349) tested positive; and (c) *Clostridium difficile* of which 13% (80/605) tested positive

The prevalence of rat‐associated pathogens is often spatially heterogeneous and may be driven by rat dispersal globally and locally. Differences in disease prevalence at regional scales (i.e., by city) are well established (Ellis et al., 1999; Kosoy & Bai, 2019; Peterson et al., 2017) and may arise through founder events, such that pathogen presence is dependent on the disease status of the individuals first introduced to an area. Indeed, the current global distribution of Norway rats has been attributed to multiple introduction events, thought to have been facilitated by human migration (Feng & Himsworth, 2014; Puckett et al., 2016). Patterns of heterogeneous pathogen prevalence are also evident at fine spatial scales (i.e., by city block) (Angley et al., 2018). For example, in Vancouver, Canada, the prevalence of the bacterial pathogen *L. interrogans* ranged from 0% to 66% by city block (Himsworth et al., 2015) (Figure 1a). Comparatively, the prevalence of *Bartonella* spp. varied from 10% to 85% by trapping location in New York City, and 0%–97% in New Orleans (Peterson et al., 2017). Similar to global movement patterns, at local scales pathogen distributions may be driven by rat dispersal and connectivity across the landscape.

Urban rats typically exhibit strong philopatry, remaining near their natal colony. Colonies of urban Norway rats can contain many individuals as the onset of sexual maturity may commence at 45 days old (Calhoun, 1963) and rats have been reported to give birth to litters of up to 11 individuals (Costa et al., 2016), although litter sizes can be larger (e.g., one litter of 21 offspring was documented by Glass, Klein, Norris, & Gardner, 2016). In some urban centers, reproduction can occur year‐round although the number of juveniles may vary by season. For example, in Vancouver, Canada, rats were found to reproduce throughout the year, but the number of juveniles was greatest in the spring and summer (Himsworth, Miller, et al., 2014). These characteristics can lead to large families of rats, with individuals occupying small home ranges about the size of a city block (reviewed in Byers, Lee, Patrick, and Himsworth (2019)). This behavioral tendency to occupy small territories, in conjunction with barriers to rat dispersal, can result in genetic discontinuities of rats across the landscape (Brouat et al., 2013; Combs, Byers, et al., 2018; Kajdacsi et al., 2013), thus potentially limiting the spread of pathogens. Indeed, such restricted connectivity has been linked to decreased spread of feline immunodeficiency virus among bobcats (*Lynx rufus*) (Kozakiewicz et al., 2020) and rabies virus among raccoons (*Procyon lotor*) (Biek, Henderson, Waller, Rupprecht, & Real, 2007). Additionally, increased contact among closely related individuals may promote unequal transmission, such that relatives are more likely to share pathogens than nonrelatives (Grear, Samuel, Scribner, Weckworth, & Langenberg, 2010; Root, Black, Calisher, Wilson, & Beaty, 2004). Although rat dispersal and family relationships may be important drivers of pathogen distributions, they remain understudied.

A lack of information regarding urban rat movement ecology is largely due to the challenges of tracking rats in real time. Traditional ecological approaches such as capture and re‐capture of marked individuals are labor and time‐intensive (Conroy & Carroll, 2009), and unequal trappability can bias movement estimates toward “trap‐happy” individuals (Byers, Lee, Bidulka, Patrick, & Himsworth, 2019). While other tools such as Global Positioning System tags enable fine‐scale monitoring over time, they remain difficult to deploy on urban rats due to issues of tag obstruction and tag removal (Byers, Lee, Donovan, Patrick, & Himsworth, 2017). Population genetic methods afford an alternative to traditional approaches by identifying closely related individuals, with accuracy improving with an increased number of genetic markers (Foroughirad, Levengood, Mann, & Frère, 2019; Premachandra, Nguyen, & Knibb, 2019). The relative locations of related individuals can be used to infer movement events. In fact, genetic approaches tend to reveal greater travel distances than suggested through traditional methods (Byers, Lee, Patrick, et al., 2019), although these patterns vary by location and sampling effort (Combs, Byers, et al., 2018). For example, genetic approaches have identified rat movement distances of up to 11.5 km in Baltimore, Maryland (Gardner‐Santana et al., 2009), and up to 536 m in New York City (Combs, Richardson, & Munshi‐South, 2018) although average movements are typically within 30–150 m (Combs, Richardson, et al., 2018; Gardner‐Santana et al., 2009). Genomics‐based approaches have also demonstrated that differences in movement can vary by sex, with males traveling further afield than females (Desvars‐Larrive et al., 2017; Kajdacsi et al., 2013) in search of mates (Glass et al., 2016). And while natal dispersal of males is common in many species of mammals (Greenwood, 1980), genetic approaches have not revealed this trend in urban Norway rats (Gardner‐Santana et al., 2009). Together, these findings suggest that patterns of relatedness vary over space and that genetic methods can provide valuable insight into movement events involved in pathogen spread or clustering.

In this study, we combine previously published disease (Himsworth et al., 2015; Himsworth, Bidulka, et al., 2013; Himsworth, Parsons, et al., 2013) and population genomic data (Combs, Byers, et al., 2018) from rats in Vancouver, Canada, to explore the role of fine‐scale genetic structure and movement in the distribution of rat‐associated pathogens. Previously, our team demonstrated spatial clustering of pathogens in this population of rats, with pathogen prevalence varying significantly by city block (Himsworth et al., 2015; Himsworth, Bidulka, et al., 2013; Himsworth, Parsons, et al., 2013) (Figure 1). While pathogen status was associated with factors such as weight, sexual maturity, and season, a significant amount of variation remained after controlling for clustering at the level of the city block (Himsworth et al., 2015; Himsworth, Bidulka, et al., 2013; Himsworth, Parsons, et al., 2013). We also found that genetic structuring varied across fine spatial scales, with some genetic clusters spanning one or several city blocks (Combs, Byers, et al., 2018). Here, we combine these datasets and use a genomics‐based pedigree inference approach to a) identify closely related individuals and infer movement events; b) compare patterns of relatedness and movement to prevalence data for pathogenic bacteria carried by rats; and c) explore the impact of family membership on an individuals' pathogen status. We hypothesized that first‐ and second‐order relatives would reside within the same city block and that these patterns of relatedness would align with the spatial clustering of pathogens. Further, we chose to evaluate rat relatedness in relation to three pathogens of public health concern (*L. interrogans*, *Bartonella tribocorum*, and *C. difficile*) as we hypothesized that family membership would contribute to pathogen status for pathogens transmitted through close contact (*L. interrogans* and *B. tribocorum*) but not for those environmentally acquired (*C. difficile*). Together, information from this study can be applied to urban rat management strategies aimed at mitigating human health risks.

## 2 METHODS

### 2.1 Ethics

This study was approved by the University of British Columbia's Animal Care Committee (A11‐0087) and adhered to national guidelines set out by the Canadian Council on Animal Care.

### 2.2 Study site

Rats were trapped in the Downtown Eastside (DTES) neighborhood of Vancouver, Canada, an area where rats are abundant (Himsworth, Jardine, Parsons, Feng, & Patrick, 2014). In Vancouver's DTES, there is considerable contact between residents and rats due in part to issues of housing affordability and availability (Byers, Cox, Lam, & Himsworth, 2019). Further, many groups living in this area are considered to be more vulnerable to health risks than are residents of Vancouver generally (City of Vancouver, 2013), which makes this area of particular concern for rat‐associated health risks. Vancouver has a moderate oceanic climate. Over this time period, the annual mean temperature was 9.6°C and annual precipitation was 81.44 mm, which were both slightly lower than the 10‐year average from 2003 to 2013 (mean annual temperature: 10.56°C; annual precipitation: 96.38 mm). The study site encompassed 43 contiguous city blocks and one site at the adjacent international shipping port on the neighborhood's northern border (N49°17′/ W123°6′). The neighborhood is densely populated with approximately 18,500 people (City of Vancouver, 2013) and is comprised of residential, commercial, and industrial buildings, many of which are in disrepair (Smith, 2000).

### 2.3 Trapping

Rats were trapped as part of a long‐term study evaluating rat disease ecology; detailed trapping methods have been published elsewhere (Himsworth, Bidulka, et al., 2013). In brief, rats were trapped from September 2011–August 2012. Each city block and the international port site was assigned randomly to a three‐week study period during the one year of trapping. We used Tomahawk Rigid Traps (Tomahawk Live Trap, Hazelhurst, USA) which were set in the alleyway bisecting each city block. Traps were prebaited for one week prior to two weeks of active trapping. We recorded the date and location of each trapped rat.

Prior to euthanasia, we collected blood via intracardiac puncture under isoflurane anesthesia. Rats were humanely euthanized via intracardiac injection with pentobarbital. At the international shipping port, rats were trapped by a collaborating pest control professional using lethal snap traps. All rats underwent a complete necropsy, with aseptic collection of the kidney, liver, and colon. Samples were stored at −80ºC prior to pathogen testing and DNA sequencing. We collected morphological data including sex, sexual maturity (scrotal testes for males, perforate vagina for females), weight (grams), and pregnancy.

For subsequent analyses, we used the von Bertalanffy (1938) growth curve equation to infer rat age in days from rat weight. This equation accounts for the nonlinear relationship between weight and age (Calhoun, 1963) and has been used previously to model rat age curves (Minter et al., 2017, 2019). Specifically: weight = a[1 – exp{–r(age – c)}], where “a” is the asymptote, “r” indicates the constant growth rate, and “c” represents the age at which maximum growth occurs. We used parameters derived from Calhoun (1963) as in Minter et al. (2019), and for pregnant females, we adjusted weight by the average difference in weight between pregnant and nonpregnant, sexually mature females (Minter et al., 2017).

### 2.4 Disease testing

All pathogen testing was completed as part of a broader epidemiological study evaluating the prevalence of rat‐associated pathogens in the DTES. For this study, we included rats that were tested for the bacterial pathogens *L. interrogans*, *Bartonella* spp., and *C. difficile*.

For *L. interrogans,* DNA was extracted from rat kidney and analyzed using a real‐time PCR that targets a 242 bp fragment of the LipL32 gene of pathogenic *Leptospira* species (Stoddard, Gee, Wilkins, McCaustland, & Hoffmaster, 2009) as outlined in Himsworth, Bidulka, et al. (2013).

For *Bartonella spp*., blood clots were cultured at the Bartonella & Rodent‐Borne Disease Laboratory, Centers for Disease Control and Prevention, Fort Collins, CO as outlined by Himsworth et al. (2015). *Bartonella* spp. were identified based on colony morphology and confirmed by PCR amplification of the citrate synthase gene (gtlA) (Bai et al., 2007; Ying, Kosoy, Maupin, Tsuchiya, & Gage, 2002).

For *C. difficile*, colon contents were cultured and identified as outlined in Himsworth, Patrick, et al. (2014). Identification of *C. difficile* was made using colony morphology and odor, Gram staining, and the presence of L‐proline aminopeptidase activity (Remel Inc., Lenexa, Kansas, USA).

### 2.5 Genetic sequencing

We used a genome‐wide single nucleotide polymorphism (SNP) dataset acquired through double digest restriction site‐associated DNA sequencing (ddRADSeq) of DNA from rat liver samples. In brief, Combs, Byers, et al. (2018) used Stacks v. 1.35 (Catchen, Hohenlohe, Bassham, Amores, & Cresko, 2013) to demultiplex sequencing reads and align them to the RNOR v.6.0 reference genome for *R. norvegicus* (Gibbs et al., 2004). Using these previously published reference‐aligned reads, Combs, Byers, et al. (2018) identified SNPs using the *pstacks*, *cstacks*, and *sstacks* pipeline from STACKS v1.35, retaining only a single SNP per RADtag. Because the parentage assignment software only requires (and allows) a limited number of loci per individual (see below), we derived a set of highly informative SNPs based on coverage, removing sites with <20× and >50× coverage using VCFtools (Danecek et al., 2011). We used PLINK 1.9 (Chang et al., 2015) to prune SNPs on the basis of linkage disequilibrium. Sliding windows of 50 SNPs (with a step of 5) were thinned using a variance inflation factor of 2. Autosomal SNPs with a minor allele frequency > 5% that were called in >85% of individuals were retained for further analysis. SNPs with excessive heterozygosity (>80%) were removed in PLINK 1.9. Our filtering criteria resulted in 1,495 SNPs genotyped in 605 individuals.

### 2.6 Pedigree inference

We identified related rats by running parentage assignment using the *Sequoia* v. 1.3.3 (Huisman, 2017) package in R (RStudio Team, 2016). Sequoia can be used effectively with relatively few loci (i.e., 500–800 SNPs; Huisman, 2019) and has been used for sibship assignment with up to 4,235 SNPs (Foroughirad et al., 2019). Simulation studies indicate that a few hundred SNPs can provide high assignment rates (99%) with low false‐positive rates (<0.1%) (Huisman, 2017). Sequoia was developed for use with SNP data and is robust to unsampled individuals. Data incorporated into the parentage analysis also include the animal's sex and birth year. Birth year is used to assist in distinguishing among relationships such as parent–offspring and full‐sibling pairs (Huisman, 2017). Given the rapid reproductive rate of rats (Feng & Himsworth, 2014) and that urban rats often live less than one year (Davis, 1953b), we defined birth years as follows: First, we estimated each rat's birth date by subtracting their computed age in days from the date of trapping. Second, based on the distribution of ages, we estimated that rats in this population reached sexual maturity at as early as 39 days as there was a division in the distribution of weights of mature and sexually immature rats at 110 g (Figure S1). Although previous estimates suggest that rats approach sexual maturity within 45–75 days for females and 45–95 days for males (Calhoun, 1963), time to maturity can vary based on resource availability (MacDonald, Mathews, & Berdoy, 1999). Finally, we calculated the number of days from the earliest estimated birth date to the latest estimated birth date and separated this time frame into 11, 39‐day intervals designated as “birth years.” Using these life history data, we identified first‐order (parent–offspring and full‐sibling) and second‐order (half‐sibling) relationships.

### 2.7 Inferring geographic and genetic distances between relatives and by sex

To characterize distances traveled, we calculated the pairwise Euclidian distance between each member of a set of relatives and categorized distances within relationship type (i.e., parent–offspring, full‐sibling, half‐sibling). We mapped relationships spatially to visualize patterns of relatedness and to identify instances where relatives were located within the same city block and in different blocks. Mapping was performed using ggplot2 in R using open‐source shape files (https://vancouver.ca/your‐government/open‐data‐catalogue.aspx) to create maps.

To identify evidence of sex‐biased dispersal, we created correlograms comparing matrices of geographic and genetic distances between pairs of male and female rats separately. Correlograms identify the extent of isolation‐by‐distance (i.e., spatial autocorrelation) for pairs of individuals at different distance classes, where variation between sexes indicate differences in dispersal intensity or mechanism (Banks & Peakall, 2012). We used the ecodist package in R, specifying distance classes of 50 m and using 1,000 permutations (Goslee & Urban, 2007).

### 2.8 Genetic relatedness and pathogen status

We used binomial generalized linear mixed models to evaluate whether rat family relationships influenced rat disease status. We designated a “family” as a group of full siblings as we hypothesized that full siblings would be most likely to have been in close proximity through nest‐sharing. This designation also prevented rat membership in multiple families (i.e., as would have occurred by including half‐siblings). For each pathogen (*L. interrogans*, *B. tribocorum*, or *C. difficile*), we created a model where the response variable was rat infection status (positive or negative), and we included rat morphometrics and capture characteristics as covariates if they were previously identified as predictors of pathogen status in this population (Himsworth et al., 2015; Himsworth, Bidulka, et al., 2013; Himsworth, Parsons, et al., 2013). As these pathogens were previously found to vary in prevalence by city block, we included city block of capture as a random effect in all models. To test whether “family” improved model fit, we included “family” as a random effect nested within city block and compared the relative fit of models with and without “family” using Akaike information criterion (AIC). Models were considered to be similarly supported if the difference between their AIC values was ≤2 (Burnham & Anderson, 2002). Modeling was performed using the package lme4 (Bates, Mächler, Bolker, & Walker, 2015) in R Studio. Additionally, we evaluated the predictive power of the “best fit” model based on AIC by calculating pseudo‐*R* ^2^ using the package MuMIn (Bartoń, 2019).

## 3 RESULTS

### 3.1 Population characteristics

A total of 685 Norway rats were trapped over the course of one year. Following filtering of genetic data, we retained 605 rats, of which 332 (55%) were male (192 mature, 139 immature, one unknown), 261 (43%) were female (129 mature, 132 immature), and 12 (2%) were of unknown sex and maturity. The number of rats tested for each rat‐associated pathogen varied. For *L. interrogans*, 535 rats were tested of which 60 (11%) were positive. For *B. tribocorum*, 349 rats were tested of which 90 (26%) were positive. For *C. difficile*, all 605 rats were tested, of which 80 (13%) were positive.

### 3.2 Pedigree inference

**FIGURE 2:**
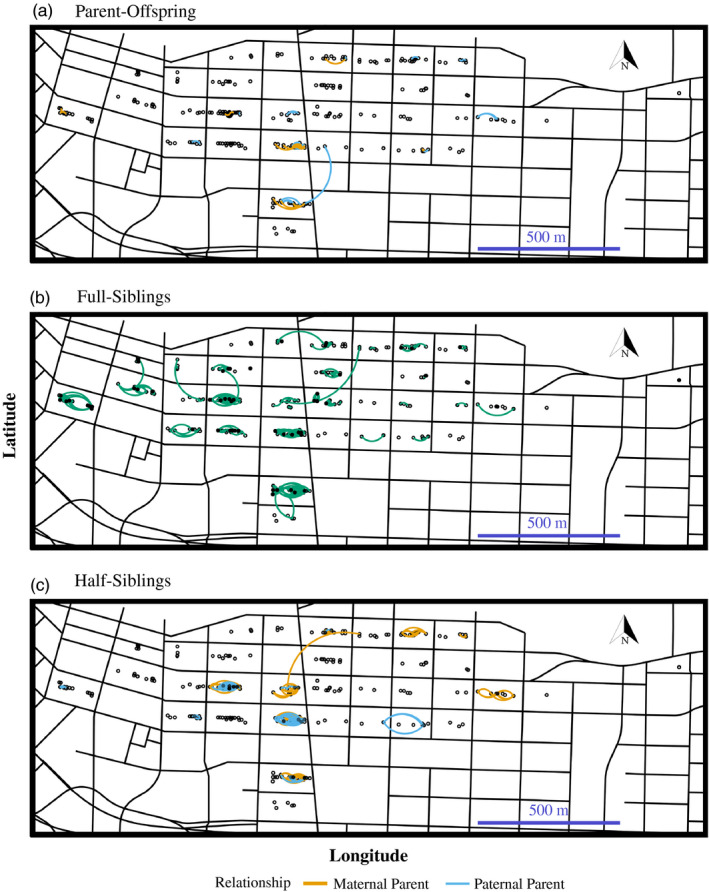
Distribution of pairs of related Norway rats (*Rattus norvegicus*) across Vancouver's Downtown Eastside neighborhood. Of 1,246 related pairs, we resolved (a) 68 parent–offspring pairs; (b) 645 full‐sibling pairs; and (c) 533 half‐sibling pairs. Thirteen pairs were associated across city blocks. Relatives captured in the same trap are identified with (•), and relatives trapped in different locations are connected by a curved line. Rats without identified relatives are indicated with (º)

Among the 605 genotyped rats included in the parentage analysis, we resolved a total of 1,246 pairs of relatives (Figure 2), 713 of which were first‐order and 533 of which were second‐order relatives. Of the first‐order relatives, we identified 68 parent–offspring pairs, including 11 dams assigned to 29 offspring and 20 sires assigned to 39 offspring (Figure 2a). Further, 72% (442/605) of rats were paired with at least one full‐sibling (Figure 2b). We identified 645 full‐sibling pairs which were grouped into 155 distinct full‐sibling “families.” These full‐sibling “families” included anywhere from 2 to 16 individuals (median = 2 individuals per family). Regarding second‐order relatives, we identified 533 half‐sibling pairs which included 314 pairs sharing a dam and 219 pairs sharing a sire (Figure 2c).

### 3.3 Geographic and genetic distances between relatives and by sex

**FIGURE 3:**
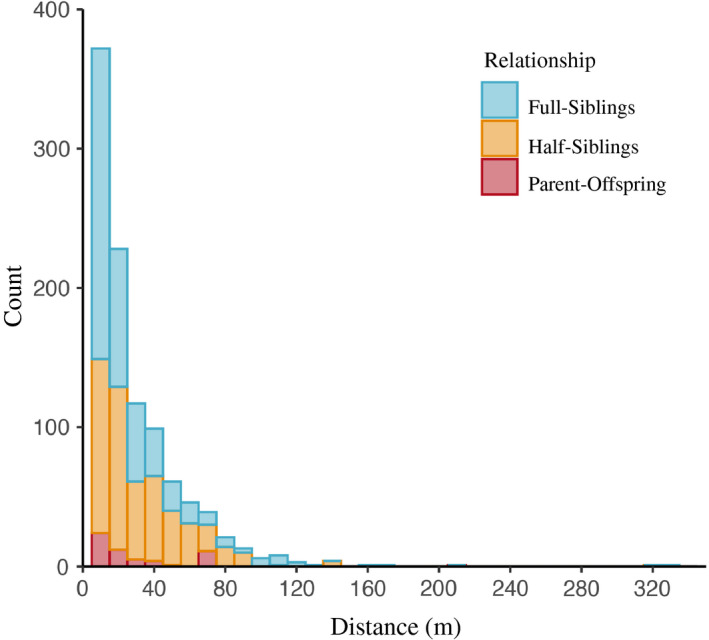
Distances between capture points of related Norway rats (*Rattus norvegicus*). Of 1,246 pairs of related rats, 75% of individuals were caught within 33 m of their relative, with 24 pairs of rats captured more than 100 m apart

**FIGURE 4:**
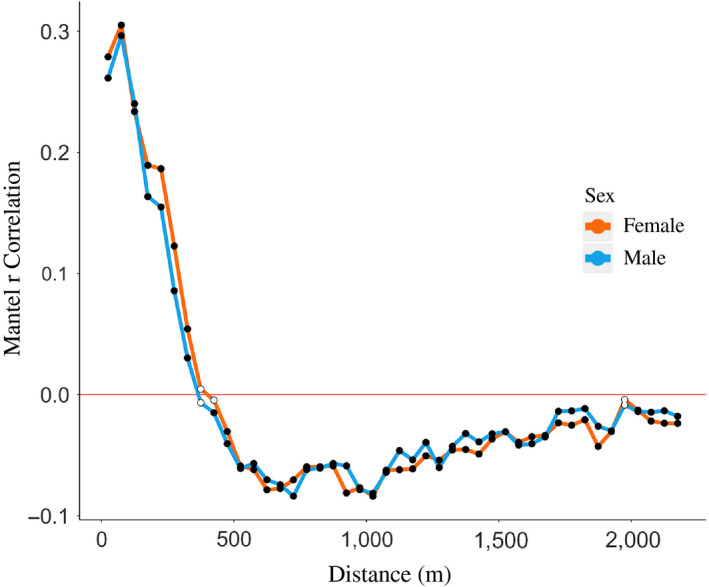
Correlogram of genetic and geographic distance for pairs of rats. The Mantel r correlation is the strength of correlation between genetic and geographic distance between pairs of male (*N* = 332) and female (*N* = 261) rats. Correlation values are denoted within each distance class of 50 m. Filled circles indicate significantly nonrandom values (α = 0.05) and open circles indicate nonsignificant relationships at that distance class

Figure 2 illustrates that the majority of rats were caught within the same city block as their relative. Of the 1,246 related pairs, 1% (13/1,246) were captured in different city blocks (one parent–offspring, nine full‐sibling, and three half‐sibling pairs). For the nine full‐sibling pairs, one pair was comprised of two juveniles, three pairs were comprised of one juvenile and one mature individual, and five pairs were comprised of two mature individuals (note that four of these pairs were part of a five‐full‐sibling family). For the three half‐sibling pairs, one pair was comprised of one juvenile and one mature individual, and the other two pairs were both comprised of mature individuals. Distances between related pairs ranged from 0 to 330 m, with 25% of relatives caught within 7 m, 50% within 16 m, and 75% within 33 m (Figure 3). Full siblings were caught in closest proximity to each other with a median distance of 10 m between pairs, while parent–offspring (median = 15.4 m) and half‐siblings (median = 22.5 m) were caught further apart. In our analysis of isolation‐by‐distance, we did not find any evidence that distance between pairs differed by sex. Patterns of isolation‐by‐distance were similar for both sexes across all distance classes (Figure 4).

### 3.4 Genetic relatedness and pathogen status

Modeling was informed by previous work on this population of rats. Specifically, we included covariates identified as informative predictors of rat pathogen status such as rat weight in the *L. interrogans* model (Himsworth, Bidulka, et al., 2013); sexual maturity and season in the *B. tribocorum* model (Himsworth et al., 2015); and weight in the *C. difficile* model (Himsworth, Patrick, et al., 2014). Table 1 depicts the parameters for the best‐fitting model for each disease. Incorporating “family” into infection models did not improve model fit. For all pathogens, models run with and without “family” as a random effect were within 2 AIC (Table 1).

**TABLE 1:** Comparison of models containing predictors of pathogen status of urban Norway rats (*Rattus norvegicus*) with and without “family” membership included as a random factor

Model		Model Includes “Family” Random Effect			Model Excludes “Family” Random Effect		Model Comparison
Pathogen	Covariates	AIC	Family Variance (Std Dev)	Block Variance (Std Dev)	AIC	Block Variance (Std Dev)	Δ AIC
*Leptospira interrogans*	Weight	219.3	0.00 (0.00)	4.99 (2.26)	217.3^a^	4.99 (2.24)	2
*Bartonella tribocorum*	Maturity Season	323.2	0.42 (0.65)	1.19 (1.09)	322^b^	1.08 (1.04)	1.2
*Clostridium difficile*	Weight	452.9	0.52 (0.72)	0.56 (0.75)	452.8^c^	0.56 (0.75)	0.1

Note

Pseudo‐*R* ^2^ for best‐fitting models:

^a^ 0.71;

^b^ 0.40;

^c^ 0.20.

At the level of the block, we identified 8 pairs of blocks that shared a pair of relatives (Figure 2). Cases where both pairs of blocks share pathogen status (i.e., both blocks either possessed affected rats or did not) were greatest for *L. interrogans* (7/8 block pairs shared pathogen status), followed by *B. tribocorum* (6/8 pairs of blocks shared pathogen status), and *C. difficile* (5/8 blocks shared pathogen status).

## 4 DISCUSSION

Understanding how rats move and interact within the urban environment is integral to informing control efforts aimed at mitigating rat‐associated impacts like the spread of zoonotic diseases. Our study is the largest parentage‐based analysis of wild rats to date and reveals fine‐scale spatial clustering of closely related individuals within 33 m of each other, with most relatives located within the same city block. These patterns of relatedness suggest very little movement and interaction of rats between neighboring city blocks which may restrict opportunities for pathogen spread.

The distinct clustering of close relatives evidenced in this study may be attributed to a combination of social and environmental barriers to movement. Urban rats are territorial (Barnett, 1963), occupying home ranges as small as 30–45 m in diameter (Davis, 1953a; Davis, Emlen, & Stokes, 1948) which is approximately 1/3 the length of city blocks in this study. While urban rats occasionally move long distances (i.e., up to 11.5 km (Gardner‐Santana et al., 2009)), long‐distance movements are rare and may be facilitated by human transport (Berthier et al., 2016; Byers, Lee, Patrick, et al., 2019). Rats often stay within their home range (Gardner‐Santana et al., 2009; Glass et al., 2016) and have been found to extend their home range and movement distances in relation to resource availability (Davis et al., 1948). It may be that rats in the DTES have little need to venture beyond their natal block due to an abundance of food and areas to burrow (Himsworth, Parsons, et al., 2014). In this regard, changes to rat density that result in fewer rats and a greater abundance of resources could alter normal rat movement patterns. Yet, in a subsequent study involving the targeted removal of rats in some city blocks, we found that rat removal did not result in a significant change in rat movement between city blocks (Byers, Lee, Himsworth, Patrick, Whitlock; in prep). It is also important to note that beyond access to resources, immigrating to a new colony can have social consequences. The territoriality of Norway rats can impede the successful integration of unknown individuals as they are often ejected by resident rats (Calhoun, 1948). Although previous work suggests that some males will travel to neighboring blocks to mate with females of a different colony (Glass et al., 2016), males will also breed with related females. For example, in Salvador, Brazil, females were more related to the sires of their offspring than would be expected (Costa et al., 2016). Therefore, it is possible that the high population density of rats in this neighborhood may limit the need of males to travel in search of mates. Indeed, we identified only one instance where a sire was captured in a different block than its offspring, and just 1% of relatives were caught in different city blocks. And while this result strongly suggests that movement among city blocks is infrequent, it is important to note that this may be an underestimate of rat movement as blocks were trapped at different times. This difference in trapping periods could bias estimates toward finding more relatives within blocks than between blocks, particularly when adjacent city blocks were trapped at separate times. However, of the eight pairs of blocks sharing relatives in this study, five were trapped during different time periods (ranging from one to eight months apart). Indeed, the study site included a total of 17 pairs of adjacent blocks that were trapped contemporaneously, and only three of these pairs shared cross‐block relatives. These patterns suggest that, although movement among city blocks may be more frequent than can be captured by this study, the majority of relatives remain within the same city block.

For all inferred movement events, it is impossible to say which individual in a pair moved, when that movement occurred, or whether gene flow is due to movement of a parent not sampled in this study. Indeed, we are only able to make inference about movements of the “trappable” population of rats, as there may be many more rats living in the sampling site that did not enter traps. Based on these findings, in at least one instance, movement occurred by a juvenile rat (i.e., both full siblings caught in different blocks were juveniles at the time of capture). In other instances of movement, it is difficult to determine both which individual moved and when they moved, as mature individuals, simply by the fact that they are older, have had more time to disperse. As approximately half of the individuals in this study are mature, a greater proportion of mature individuals may have revealed greater levels of gene flow. Our results suggest that males and females contribute similarly to gene flow, with little evidence for sex‐biased dispersal. These findings align with studies in Baltimore and New York City, USA (Combs, Richardson, et al., 2018; Gardner‐Santana et al., 2009), although sex‐biased dispersal has been reported in both Salvador, Brazil and Hauts‐de‐Seine, France (Desvars‐Larrive, Baldi, Walter, Zink, & Walzer, 2018; Kajdacsi et al., 2013). Beyond social barriers, environmental features may also impede rat movement. Landscape features such as high‐traffic roadways, waterways, and areas with fewer resources have been found to align with restricted gene flow, suggesting that these environmental characteristics can pose a barrier to movement (Combs, Byers, et al., 2018; Richardson et al., 2017). In fact, Combs, Byers, et al. (2018) indicated that roadways in this study were likely barriers to dispersal, although in some nonurban contexts roads may facilitate movement through commercial transport (Berthier et al., 2016). Overall, the minimal connectivity of rats among city blocks explains the high levels of inbreeding previously reported in this population (F_IS_ ranging from 0.06–0.28) (Combs, Byers, et al., 2018). As urban centers densify and land use changes (i.e., through emphasis on “greening” city spaces (i.e., Goddard, Dougill, and Benton (2010); Lovell and Taylor (2013)), these patterns are likely to change in response to altered segregation of rat colonies with implications for pathogen spread.

The patterns of limited connectivity among city blocks in Vancouver align with heterogeneous patterns of pathogen prevalence (Himsworth et al., 2015; Himsworth, Bidulka, et al., 2013; Himsworth, Parsons, et al., 2013). We find that in instances where there were cross‐block relatives, these pairs of blocks often shared pathogen status (i.e., blocks either both had affected rats, or they did not). These patterns were most striking for *L. interrogans* and *B. tribocorum*, where seven of eight pairs of connected blocks and six of eight pairs of connected blocks shared pathogen status respectively. While it is difficult to ascertain whether these patterns are driven by rat movement, connectivity through movement may allow for the spread of some pathogens due to aggressive interactions between the immigrating individual and members of the established colony (Calhoun, 1948). These interactions would be particularly important for facilitating transmission of pathogens such as *L. interrogans* and *B. tribocorum*, as they are transmitted through contact with rats and their parasites. Similar trends might be excepted for other rat‐associated pathogens transmitted through biting and aggressive interactions (i.e., *Streptobacillus moniliformis*) close contact with urine and feces (i.e., Seoul hantavirus) and vectors such as fleas (i.e., *R. typhi*) (Himsworth, Parsons, et al., 2013) yet these relationships remain to be studied. Importantly, these rat‐associated pathogens are not associated with illness in rats (Himsworth, Parsons, et al., 2013) and therefore are not known to differentially impact rat movement. However, one study evaluating whether infection with *L. interrogans* influenced rat trappability (which could in turn affect movement estimates) found no association between *L. interrogans* carriage and trappability (Byers, Lee, Bidulka, et al., 2019). Therefore, links between movement and pathogen distributions will be highly dependent on pathogen ecology.

Within city blocks, fine spatial structuring appears to be less important in determining pathogen status than block‐level associations. As full siblings share a nest until they begin free‐roaming at as early as 25 days old (Calhoun, 1963), we hypothesized that full siblings would be more likely to share pathogen status. However, membership in a full‐sibling “family” did not appear to explain pathogen status. While this result was expected for *C. difficile*, a pathogen thought to be transmitted among rats through environmental contamination (Himsworth, Patrick, et al., 2014), this result was unexpected for *B. tribocorum* and *L. interrogans* carriage. Specifically, because *Bartonella* spp. is transmitted among rats through contact with their fleas, which reside both on rodents and in their nests (Krasnov, Khokhlova, & Shenbrot, 2004), where rats regularly maintain close physical contact (Barnett, 1963), we expected family membership to account for some of the variation in *B. tribocorum*, status. However, as multiple paternity is common in Norway rats (Costa et al., 2016; Glass et al., 2016), individuals are also likely to share the nest with half‐siblings. Therefore, a more extensive designation of “family” which includes half‐siblings may better elucidate the role of nest‐sharing in *B. tribocorum* status. We also hypothesized that “family” would in part explain *L. interrogans* carriage as it is transmitted through contact with affected rat urine (Costa, Wunder, et al., 2015) and may also be transmitted through social interactions such as biting (Minter et al., 2019). As previous work suggests that most rats in this area acquire *L. interrogans* after leaving the nest (Minter et al., 2019), contact with urine‐contaminated water in alleyways may be a more important source of infection than contact with urine near the nest. Importantly, these trends will vary by city. By comparison, rats in Salvador, Brazil, were found to acquire *L. interrogans* prior to leaving the nest, suggesting that variations between these two urban environment alter patterns and timing of pathogen acquisition (Minter et al., 2019). Further, as biting often occurs through fighting and contests for dominance (Barnett, 1963), it is possible that these interactions occur at a similar frequency among closely and more distantly related rats. To ascertain the social interactions of urban rats, further rat behavioral work is necessary, with the last in‐depth studies on urban rat behavior occurring over 50 years ago (i.e., Davis and Christian (1956); Calhoun (1963)). Overall, our results suggest that block‐level associations are more powerful for explaining patterns of pathogen prevalence than are closer full‐sibling relationships; however, a more extensive consideration of how rats interact with each other within and between colonies is needed to resolve these dynamics.

The relationship between rat genetic structure and pathogen prevalence provides an opportunity for pest control professionals seeking to mitigate rat‐associated health risks. First, the clustering of close relatives within city blocks suggests that, in the short term, the “city block” may serve as an appropriate eradication unit with barriers such as roadways serving as natural borders to management (Combs, Byers, Himsworth, & Munshi‐South, 2019). Second, the highly heterogeneous distributions of rat‐associated pathogens such as *L. interrogans* and *B. tribocorum* can allow management approaches to prioritize blocks with high pathogen prevalence. Such targeting can be used to address disease prevention. However, it is important to note that even within a neighborhood, connectivity among city blocks can vary. Indeed, we find more inferred movement events in the northern area of the study site, while previous work has demonstrated that genetic clusters of closely related individuals can span several city blocks (Combs, Byers, et al., 2018). Further, underground infrastructure such as sewers may provide opportunities for movement among blocks otherwise segregated by roadways, and therefore, these avenues of connectivity and pathogen spread must also be considered. The potential for the reinvasion of managed areas (Davis, 1953b; Hansen, Hughes, Byrom, & Banks, 2020) in combination with population rebounds attributed to the survival and reproduction of rats following an eradication campaign (Barnett & Bathard, 1953; Hacker et al., 2016), necessitates more broadly applied, long‐term approaches to address neighborhood‐level infestation. To support long‐term population reduction, an increasing number of studies articulate the need to target the underlying habitat features which promote rat infestations such as access to food and areas to burrow (Corrigan, 2011; Lambert, Quy, Smith, & Cowan, 2008; Singleton, Leirs, Hinds, & Zhang, 1999).

## 5 CONCLUSIONS

Despite their infamy as long‐distance travelers, we contribute to the growing evidence that urban Norway rat movement is highly localized. We demonstrate that even within city blocks, related rats are aggregated often within 33 m. These clusters of closely related individuals align with heterogenous patterns of pathogen prevalence, particularly for pathogens transmitted through close contact with rat excreta and ectoparasites such as *L. interrogan* *s* and *B. tribocorum*. Management approaches, particularly those facing resource limitation, may benefit from targeting city blocks with high pathogen prevalence in order to address the most pertinent public health issues. In instances where the scope of management efforts is applied to at least the level of the city block, it may minimize pathogen spread among remaining rats and between blocks.

## CONFLICT OF INTEREST

None declared.

## Supporting information

Fig S1

## Data Availability

Data for this study are available on the Dryad Digital Repository https://doi.org/10.5061/dryad.t4b8gthzt.
