# Supplementary material for: Using genetic relatedness to understand heterogeneous distributions of urban rat‐associated pathogens
Source: Evol Appl. 2020 Jul 23;14(1):198–209. doi: 10.1111/eva.13049 (PMC7819557; doi:10.1111/eva.13049)
Supplement: Supplementary file 1 — Fig S1 [file EVA-14-198-s001.pdf]

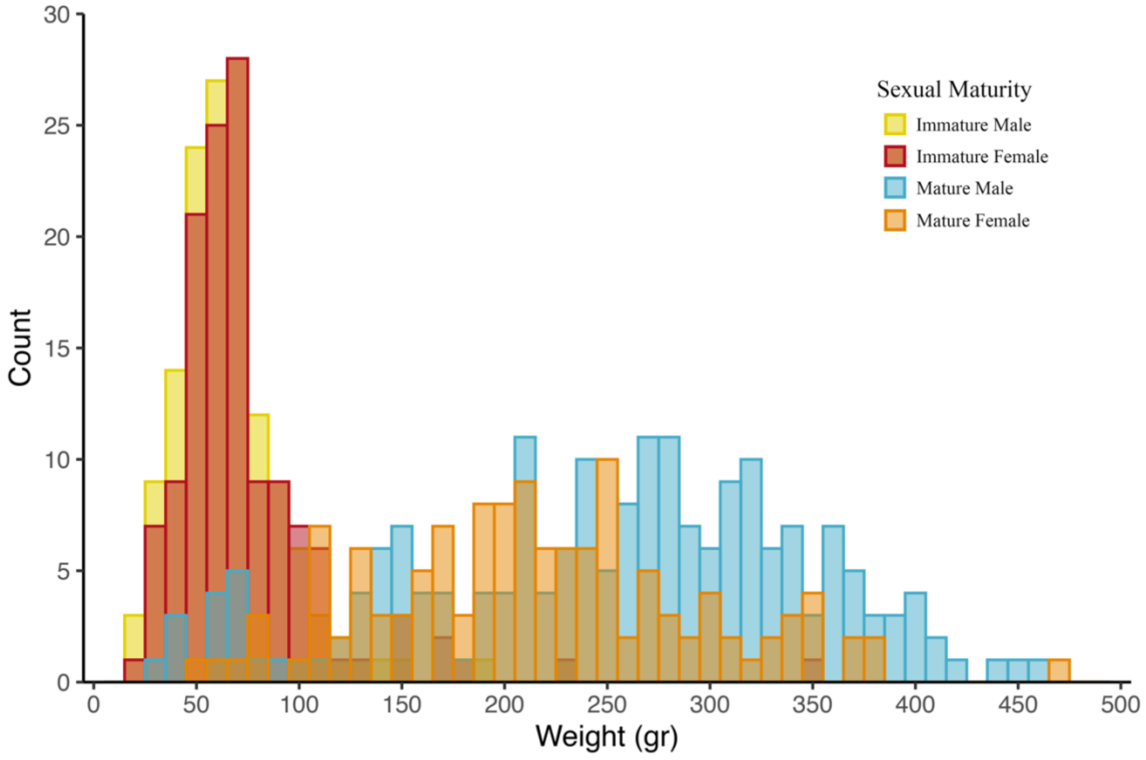

Supplementary Figure 1. Distribution of weights for Norway rats (*Rattus norvegicus*) by sex and sexual maturity. Weight in grams is displayed for rats identified as immature males (N = 139), immature females (N = 132), mature males (N = 192), and mature females (N = 129). Weights for pregnant females were adjusted by the average difference between pregnant and non-pregnant, sexually mature females.
